# Advances in Nucleic Acid Assays for Infectious Disease: The Role of Microfluidic Technology

**DOI:** 10.3390/molecules29112417

**Published:** 2024-05-21

**Authors:** Yiran Wang, Jingwei Chen, Zhijin Yang, Xuanyu Wang, Yule Zhang, Mengya Chen, Zizhen Ming, Kaihuan Zhang, Dawei Zhang, Lulu Zheng

**Affiliations:** 1 Engineering Research Center of Optical Instrument and System, The Ministry of Education, Shanghai Key Laboratory of Modern Optical System, University of Shanghai for Science and Technology, Shanghai 200093, China; 2 Shanghai Institute of Immunology, Department of Immunology and Microbiology, Shanghai Jiao Tong University School of Medicine, Shanghai 200025, China; 3 2020 X-Lab, Shanghai Institute of Microsystem and Information Technology, Chinese Academy of Sciences, Shanghai 200050, China; 4 Shanghai Engineering Research Center of Environmental Biosafety Instruments and Equipment, University of Shanghai for Science and Technology, Shanghai 200093, China; 5 Shanghai Institute of Intelligent Science and Technology, Tongji University, Shanghai 200092, China

**Keywords:** microfluidic system, nucleic acid assay, infectious disease, high throughput, rapid diagnosis

## Abstract

Within the fields of infectious disease diagnostics, microfluidic-based integrated technology systems have become a vital technology in enhancing the rapidity, accuracy, and portability of pathogen detection. These systems synergize microfluidic techniques with advanced molecular biology methods, including reverse transcription polymerase chain reaction (RT-PCR), loop-mediated isothermal amplification (LAMP), and clustered regularly interspaced short palindromic repeats (CRISPR), have been successfully used to identify a diverse array of pathogens, including COVID-19, Ebola, Zika, and dengue fever. This review outlines the advances in pathogen detection, attributing them to the integration of microfluidic technology with traditional molecular biology methods and smartphone- and paper-based diagnostic assays. The cutting-edge diagnostic technologies are of critical importance for disease prevention and epidemic surveillance. Looking ahead, research is expected to focus on increasing detection sensitivity, streamlining testing processes, reducing costs, and enhancing the capability for remote data sharing. These improvements aim to achieve broader coverage and quicker response mechanisms, thereby constructing a more robust defense for global public health security.

## 1. Introduction

In recent years, pandemics caused by pathogenic microorganisms, including the novel coronavirus (COVID-19), Zika virus, dengue fever, and Ebola virus (EBOV), have caused significant disruption and damage upon human societies [1,2]. Data from China and Italy suggest a case-fatality rate of 2.3% in patients with COVID-19, with Western Europe and the US being particularly severely affected [3]. The Zika virus has also become the focus of a public health emergency, previously primarily limited to sporadic cases in Africa and Asia [4]. It has been reported that more than two million people worldwide have been infected [5]. Additionally, the Ebola virus is mainly endemic in West Africa and is known for its high mortality rate. The 2014 outbreak was particularly severe, resulting in 28,646 infections and 11,323 deaths [6]. Dengue fever is also a disease of significant public health concern. According to the World Health Organization, dengue fever is one of the leading causes of death among children in South American countries, and more than 2.5 billion people worldwide are at risk of contracting the disease [7]. These outbreaks have not only led to the deaths of millions but have also imposed long-term negative impacts on the global economy, public health systems, and the fabric of daily life [8]. Concurrently, these emergencies have accentuated the critical need for the rapid and precise identification of pathogenic microorganisms. Rapid diagnostic technologies can not only identify infected individuals in a timely manner, breaking the chains of transmission, but also enable healthcare providers to make expedited treatment decisions and optimize resource allocation [9]. Thus, effectively controlling the spread of disease reduces the societal and economic burden [10]. Consequently, the development and application of efficient pathogen detection tools are urgent for strengthening global strategies for disease prevention, control, and response [11].

Nucleic acid assays are recognized as the gold standard for detecting viruses, as they enable direct measurement of parts of the viral genome. The nucleic acids of the virus are primarily present in the nasopharyngeal secretions of those infected, thus making these tests crucial in preventing the transmission of virus across populations and communities [12]. Traditional methods include quantitative RT-PCR [13,14], nucleic acid hybridization techniques, DNA sequencing technologies, LAMP, isothermal amplification reactions (ITA) [15], CRISPR-Cas technology, and so on [16].

However, microfluidic devices enhance nucleic acid amplification efficiency through the encapsulation of reagents systems within sealed chips, effectively reducing the risk of external aerosol contamination. The microscale structure and rapid thermal transfer characteristics of these devices not only decrease the volume of reaction needed but also shorten the thermal cycling time [17,18,19]. Furthermore, microfluidic technology allows for the integration of laboratory platforms, combining the strengths of traditional molecular methods and other techniques into a single operation, avoiding cumbersome manual processes [20,21]. Up to now, microfluidic systems based on nucleic acid assays have made significant progress, with diversified device designs and detection methods. However, the architecture of the general system design remains similar. These systems are primarily composed of elements such as micro-channels, micro-reaction chambers, micro-valves, micro-pumps, micro-heaters, temperature sensors, and detectors, as shown in the graphical abstract. Compared with conventional clinical methods, the microfluidic-based detection system has a shorter average detection time [22], higher throughput [23], and lower cost [24], significantly enhancing the availability of these approaches. Hence, microfluidic technologies have been widely applied for the detection of various viruses, such as the SARS-CoV-2, Ebola, and Zika viruses, especially exhibiting significant advantages in the rapid diagnosis and monitoring of epidemic viruses. In this review, we summarize nucleic acid assays combined with microfluidic systems. Microfluidic technology, known for its efficiency and accuracy, has been widely adopted for nucleic acid testing of various biological samples. These samples include saliva, nasopharyngeal swabs, blood, urine, and extracellular vesicles. Saliva samples, due to their convenient and non-invasive collection, are commonly used for rapid screening and early disease diagnosis [25,26]. Nasopharyngeal swabs, as the standard testing material for respiratory diseases like COVID-19, effectively collect secretions from the upper respiratory tract [26]. Blood, rich in biological markers, is frequently used to detect molecular indicators of systemic diseases [27]. Urine testing offers a non-invasive method suitable for long-term monitoring and diagnosing of metabolic abnormalities [28]. Additionally, extracellular vesicles, as critical mediators of cell-to-cell communication, contain nucleic acid information that can reveal the molecular mechanisms of diseases [29]. Utilizing microfluidic platforms for the extraction and analysis of nucleic acids from these various sample types not only enhances the sensitivity and specificity of the tests but also significantly reduces reagent usage and processing time. First, we discuss the application of microfluidic systems to optimize the conventional detection methods, including PCR, LAMP, and CRISPR. Next, we explore the application of emerging technologies in this field, such as smartphone-based and paper-based testing. Ultimately, our aim is to inspire and encourage further research and advancement of microfluidic technology for nucleic acid assays by summarizing recent advancements.

## 2. The Application of PCR Technology in Microfluidic Platforms

**Figure 1:**
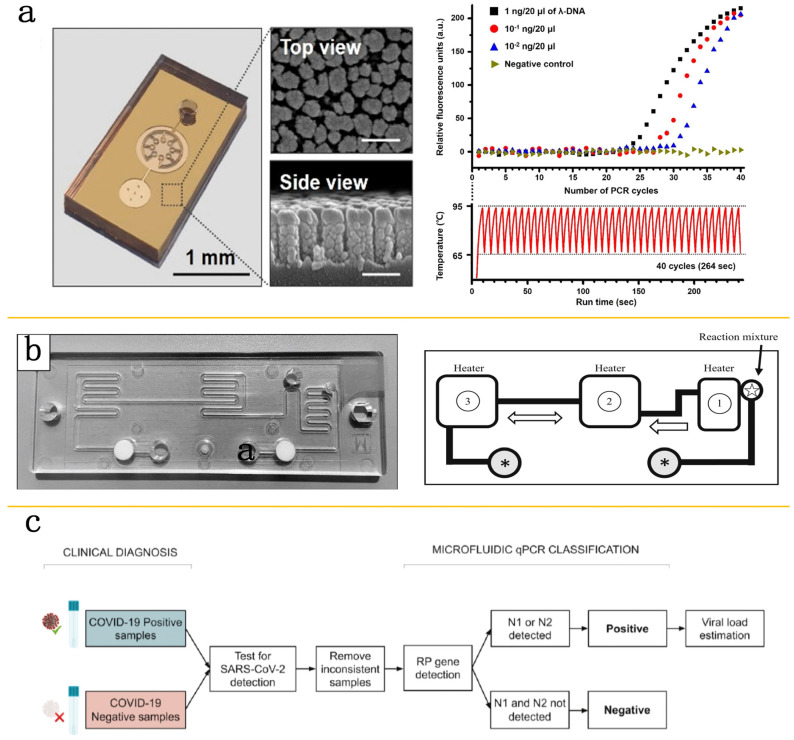
Microfluidic system combined with PCR technology. (**a**) Image of a PF-PCR chip (dimensions: 14 × 26 × 4 mm) (**left**) accompanied by cross-sectional scanning electron microscopy (SEM) images of PNA (**middle**). Amplification curves and temperature profiles of lambda DNA (λ-DNA) during a 40-cycle, two-step nano-plasmonic PCR ranging from 65 to 95°, covering 264 s, based on varying initial concentrations of λ-DNA (**right**). Reproduced with permission [30]. Copyright 2021 American Chemical Society. (**b**) The fast quantitative RT-qPCR microfluidic setup (**left**) and the schematic representation of fluid dynamics on a single-use microfluidic chip (**right**). Reproduced with permission [31]. Copyright 2020 Elsevier Ltd. (**c**) Flowchart for reanalysis of clinical samples (SARS-CoV-2-positive and -negative). Reproduced with permission [35]. Copyright 2020 MDPI, Basel, Switzerland.

The application of microfluidic-platform-integrated PCR technology has demonstrated excellent efficiency and sensitivity in the detection of viruses. This synergy of precision manipulation of the fluid in the microfluidic chip with the robust amplification power of PCR enables rapid extraction, reverse transcription, and amplification of viral RNA. It significantly shortens detection time and improves sensitivity and accuracy, especially in responding to urgent public health events like the COVID-19 pandemic. For COVID-19 detection, a microfluidic chip equipped with glass nanopillar arrays could realize the automation of sample loading and bubble-free PCR reactions. This chip facilitates ultrafast photo-thermal cycling through nanopillar arrays, greatly reducing the reaction time. The obtained results indicated that plasmids which could express the SARS-CoV-2 envelope protein can be amplified within 306 s, with an amplification efficiency exceeding 91% [30] (Figure 1a). Another PCR system integrates heaters and thermal cyclers with micro blowers, achieving high-speed PCR solution exchange and completing the amplification of specific genes within a mere 15 min [31] (Figure 1b). These advances indicate the tremendous potential of microfluidic technology in enhancing experimental efficiency and reducing external contamination. RT-PCR technology has acceptable sensitivity for early infection detection. RT-PCR begins by converting the virus’s RNA into complementary DNA (cDNA), followed by Taq DNA polymerase-mediated amplification of the cDNA. Finally, the process completes with the quantitative analysis of the viral load. However, it is prone to resulting in false positives or false negatives [32,33,34]. To address these issues, the research by Xie and colleagues has led to the creation of a novel three-stage microfluidic system that integrates both reverse transcription and pre-amplification, significantly increasing the detection capacity for low-viral-load samples and solving the false-negative issue. This method is termed nano-level PCR technology, which can detect viral quantities as low as 1 copy/μL [35] (Figure 1c). Additionally, Fassy et al. have added a pre-amplification step, enabling nucleic acid detection in each reaction in nasopharyngeal swab samples [36]. Furthermore, centrifugal microfluidic technology, known for its integrality, simplicity, and the ability to be driven by a single-spindle motor across hundreds of independent units, has been widely employed in the amplification reactions of SARS-CoV-2 [19,37,38]. The progress of these technologies provides vital means to reduce false negative or false positive results, which is important for enhancing diagnostic accuracy for SARS-CoV-2.

RT-PCR is considered the gold standard for diagnoses like pneumonia. While RT-PCR stands out for its sensitivity, precision, and specificity in identifying viruses, it still has limitations, such as sample storage issues [39,40], contamination from external aerosols [38], long waiting periods [40], the need for large equipment [41], and professional requirements for the operators [39,41,42].

## 3. Isothermal Amplification Techniques on Microfluidic Platforms

**Figure 2:**
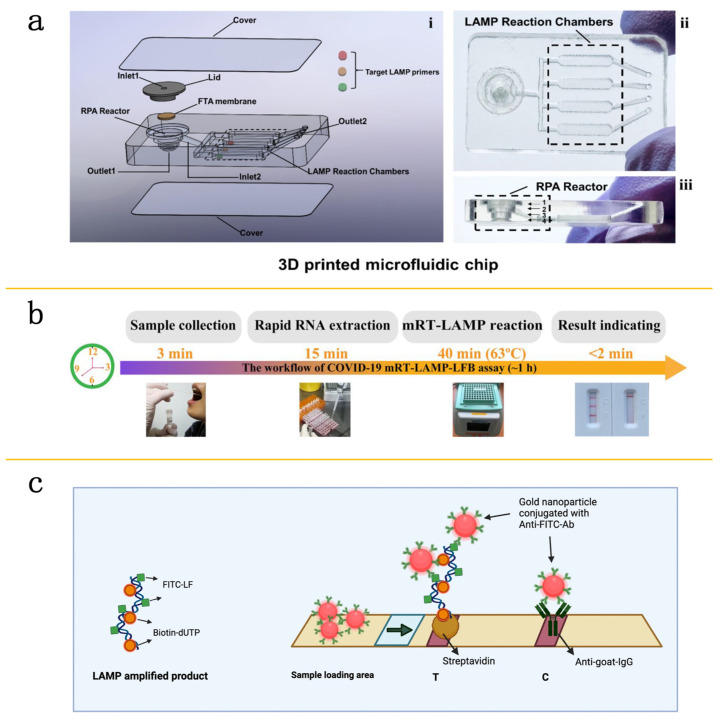
Application of isothermal amplification technique in microfluidic testing. (**a**) Schematic representation of the LAMP reaction chamber in microfluidic chip: (**i**) disassembled view; (**ii**) overhead view; (**iii**) lateral view. Reproduced with permission [44]. Copyright 2021 Elsevier B.V. (**b**) The process of the mRT-LAMP-LFB assay consists of four main steps: collecting the sample (3 min), performing rapid RNA extraction (15 min), conducting the mRT-LAMP reaction (40 min), and reporting the results (less than 2 min). This entire RT-LAMP-LFB diagnostic test can be completed in under 60 min. Reproduced with permission [45]. Copyright 2020 Elsevier B.V. (**c**) Schematic of the visual readout of the LAMP product using a lateral flow assay. During amplification, molecules of biotin and FITC are incorporated into the LAMP product. A test line (T) becomes visible on the assay strip only if the LAMP product is recognized by GNP-Anti-FITC-Ab. Meanwhile, a control line (C) serves as an internal check to confirm the assay’s validity. Reproduced with permission [46]. Copyright 2022 Springer Nature.

Isothermal amplification reactions do not require complex temperature cycling, which simplifies the experimental steps compared to RT-PCR [26]. The LAMP technique has received extensive attention for its absolute sensitivity, rapid response, ease of use, and straightforward readouts [43]. The number of SARS-CoV-2 microfluidic detection methods developed according to LAMP technology is increasing. These methods allow for sensitive, multiplexed colorimetric assays through the integration of nucleic acid isolation, dual-step isothermal amplification, and colorimetric analysis in a single microchip. For instance, researchers have effectively detected SARS-CoV-2 in wastewater samples within an hour, with detection limits as low as 100 genome equivalents (GE)/mL and 500 colony-forming units (CFU)/mL [44] (Figure 2a). Zhu and colleagues have combined multiplex RT-LAMP with a nanoparticle-based lateral flow biosensor for SARS-CoV-2 detection and N genes with a detection limit of 12 copies, with both sensitivity and specificity reaching 100% [45] (Figure 2b). To further improve sensitivity and reduce detection time, a lateral flow RT-LAMP method utilizing enzyme-linked biotinylated dUTP has been developed, reducing the detection time to 15 min [46] (Figure 2c).

Additionally, other isothermal amplification techniques like Nucleic Acid Sequence-Based Amplification (NASBA) [47] and recombinase polymerase amplification (RPA) [48] have also been utilized in microfluidic chip experiments to detect SARS-CoV-2 pathogens. For instance, Yeh et al. have demonstrated an all-in-one microfluidic diagnostic device that provides quantitative analysis of nucleic acids straight from blood without the need for preparation steps. Utilizing the RPA method, this device can detect Methicillin-Resistant Staphylococcus aureus (MRSA) DNA (between 10 and 10^5^ copies per microliter of blood) within approximately 30 min [49]. These advancements show the potential of isothermal amplification technologies in simplifying procedures and enhancing detection sensitivity.

In the detection of Ebola virus, cutting-edge microfluidic assays combined with RT-LAMP have demonstrated the advantages of high throughput, rapidity, and high sensitivity. Recently, Lin et al. introduced a disc-chip utilizing the RT-LAMP method capable of detecting four different strains of EBOV. The system can detect SUDV, EBOV, BDBV, and TAFV as low as 1 copy/μL, 100 copies/μL, 1000 copies/μL, and 10 copies/μL, respectively, within a 50 min timeframe [22].

**Figure 3:**
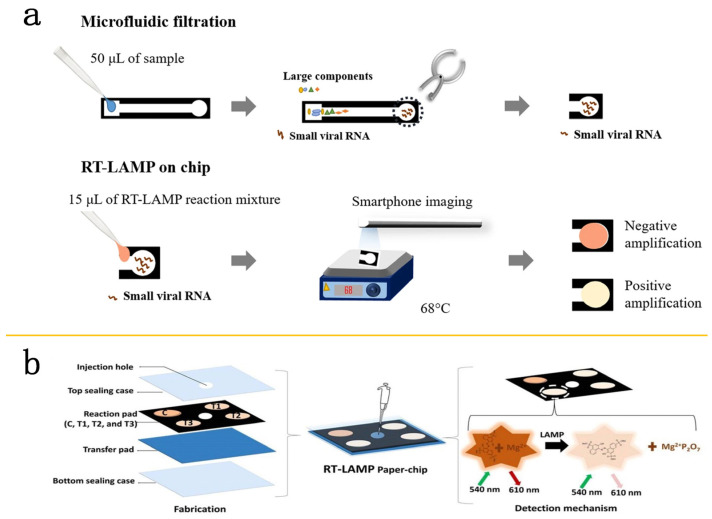
The detection technology of isothermal amplification combined with microfluidics. (**a**) Workflow of the paper-based RT-LAMP assay. Samples spiked with ZIKV were placed onto the loading zone of a paper-based microfluidic device and moved through the channel by capillary action. The circular end (detection zone) of the paper was then cut off. Following this, the RT-LAMP reaction mix was applied to it and the sample was heated on a hot plate at 68 °C for up to 40 min to facilitate amplification. Reproduced with permission [53]. Copyright 2018 Springer Nature. (**b**) Diagrammatic illustration of the RT-LAMP paper chip designed for concurrent amplification (**left**) and detection of various viral RNAs (**right**). Reproduced with permission [54]. Copyright 2019 Elsevier B.V.

On the other hand, microfluidic tools have been effective in early Zika diagnosis, with nucleic acid-based microfluidic chips playing a crucial role in Zika detection [50]. For instance, Severino demonstrated a one-step RT-LAMP platform used for Zika detection. RT-LAMP detection offers high specificity for Zika, with a sensitivity that is 10,000 times higher than qRT-PCR, 100% sensitivity, 91.18% specificity, and an accuracy of 95.24% [51]. Ganguli and colleagues designed a microfluidic chip integrated with a smartphone for RT-LAMP technology that can accurately detect Zika and other viruses. The system has a minimum LOD (1.56 × 10^5^ PFU/mL) in blood samples within only 35 min [52]. Kaarj et al. took this step further by combining a paper-based microfluidic chip with a smartphone for enhanced RT-LAMP testing, designed for detecting Zika RNA. This system is characterized by its high sensitivity and is able to detect 1 copy/μL of viral RNA within 15 min, although this innovation requires an extra purification step [53] (Figure 3a). Batule et al. have developed a dual-stage paper-based chip designed for RNA extraction and subsequent RT-LAMP, capable of detecting 10 copies of the virus in serum within 1 h [54] (Figure 3b).

Additionally, Mendes et al. have engineered a compact and disposable microfluidic device for the detection of dengue virus (DENV), which was transmitted through four different serotypes, including DENV1, DENV2, DENV3, and DENV4 [55,56]. Utilizing RT-LAMP technology, this device is capable of identifying the DENV4 strain in authentic serum samples in a single, streamlined step. The device obtained a Limit of Detection (LOD) of 0.8 fg/μL within 2 min [57].

LAMP is a stable and powerful nucleic acid amplification technique that utilizes chain replacement activity in an isothermal environment while eliminating the complex thermal cycle process to improve the limitations of PCR. It is considered a promising technology for the next generation of POC devices due to its excellent sensitivity, rapidity, elasticity, specificity, and portability [58]. However, LAMP also has certain limitations. Firstly, due to inadequate digestion of amplified products, it faces challenges in multiplex detection [59]. Secondly, the uncertainty of primers may lead to the generation of self-amplifying products, resulting in false positive results [60].

## 4. Application of CRISPR-Cas Technology in Microfluidic Platforms

**Figure 4:**
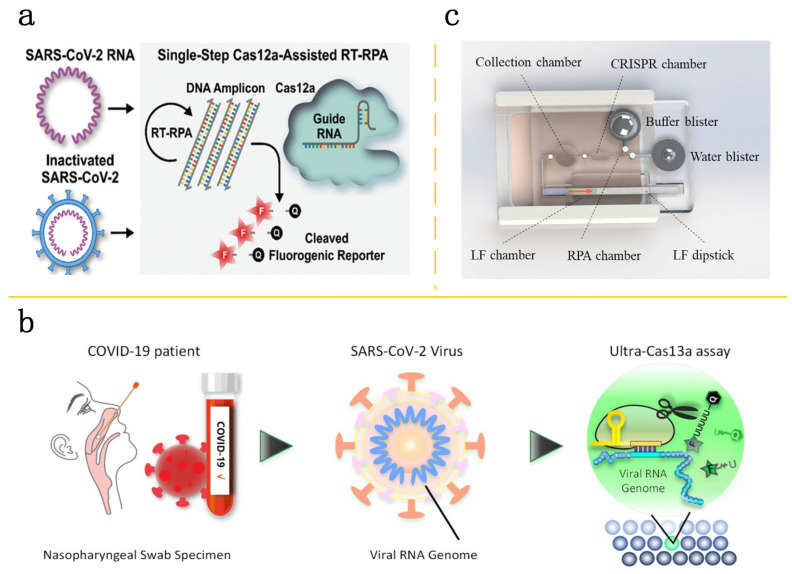
Combination of CRISPR-Cas with microfluidic system. (**a**) A single-step CRISPR/Cas12a-assisted reverse transcription recombinase polymerase amplification (RT-RPA) assay can detect SARS-CoV-2 RNA and inactivated SARS-CoV-2 virus. This method converts RNA targets into DNA amplicons, which then trigger the Cas12a-based cleavage of fluorogenic reporters in one streamlined process. Reproduced with permission [61]. Copyright 2021 Wiley-VCH GmbH. (**b**) As designed, crRNAs recognize target regions within a viral RNA genome in a droplet, and this interaction initiates the trans-cleavage of quenched fluorescent reporters. Consequently, the droplet “lights up”, signaling the presence of the target of interest. Reproduced with permission [64]. Copyright 2020 American Chemical Society. (**c**) The microfluidic chip is paired with a heater case powered by a hand warmer, which generates chemical heat. LF stands for lateral flow. Reproduced with permission [65]. Copyright 2021 Elsevier B.V.

As the application of CRISPR-Cas technology expands for infectious pathogen detection, an increasing number of studies have validated its advantages as a detection tool. These advantages include the ability to perform rapid and precise detection and to operate at lower reaction temperatures. The integration of commercial microfluidics-based digital chips with a CRISPR/Cas12a-enhanced RT-PCR technique allows for the qualitative identification of pathogens in as little as 15 min and quantitative assessments in approximately 30 min. The method demonstrates a robust signal-to-noise ratio and excellent sensitivity, capable of identifying SARS-CoV-2 concentrations as low as 1 and 20 GE/mL for inactivated virus samples [61] (Figure 4a). Specifically, when combined with microfluidic technology, the diagnostic capabilities of these techniques are significantly enhanced. In the field of COVID-19 testing, a platform that integrates CRISPR-based diagnostics with commercialized microfluidics technology has not only simplified the workflow for clinical use but has also been tested on 525 patient samples in laboratory settings and 166 samples in clinical contexts. Moreover, this platform has the capacity to detect six SARS-CoV-2 variants, including Omicron, through the examination of over two thousand patient samples, facilitating the high-capacity surveillance of various of viruses and their mutations [62]. Furthermore, advancements in selective ion focusing through precise electric field gradients on microfluidic platforms enable the purification of the target RNA automatically from nasopharyngeal samples. Combining LAMP and CRISPR-Cas12 detection technologies with the SARS-CoV-2 testing achieves results within 35 min [63]. In the application of microfluidic droplet technology, by utilizing a Cas13a catalytic system triggered by RNA within cell-sized droplet reactors, both the concentration of the target RNA and the reporter gene are enhanced. This method increases sensitivity by over 10,000 times compared to traditional Cas13a assays, achieving absolute digital single-molecule RNA quantification [64] (Figure 4b). Additionally, in 2022, Li and associates pioneered a microfluidic system that combines isothermal amplification with CRISPR-based cleavage and lateral flow assays to allow for visual detection free from contamination risks. The sensitivity and specificity of this method for COVID-19 detection were 94.1% and 100%, respectively [65] (Figure 4c). Broughton and colleagues reported the creation of a fast (under 40 min), straightforward, and precise CRISPR-Cas12-based lateral flow test to identify SARS-CoV-2 in RNA extracts from respiratory swabs. This assay demonstrated a 95% positive predictive agreement and a 100% negative predictive agreement [66].

**Figure 5:**
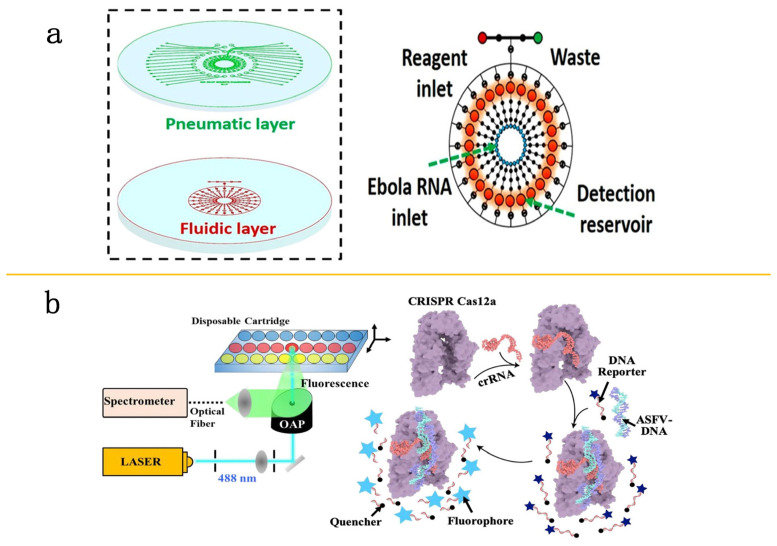
Application of CRISPR-Cas technology in microfluidic detection. (**a**) An automated POC system for EBOV RNA testing. Ebola target RNA is introduced into the detection reservoir, where it undergoes a reaction with Cas13a-crRNA. Reproduced with permission [67]. Copyright 2019 American Chemical Society. (**b**) Schematic representation of the fluorescence sensing and the CRISPR/Cas12a detection mechanism. CRISPR/Cas12a interacts with crRNA and DNA, forming the Cas12a/crRNA complex, which then cleaves a single-stranded DNA probe to facilitate fluorescence detection. Reproduced with permission [68]. Copyright 2020 Elsevier B.V.

In the detection fields for Ebola virus, Qin and collogues have designed a POC system for automatic EBOV RNA detection. This system proceeds with immediate measurement of Cas13a’s nonspecific cleavage products using an integrated fluorometer that is compact in size, facilitating on-site diagnostics. The system achieves a sensitivity threshold of 20 PFU/mL within 5 min; characterized by its speed, no need for amplification, ease of use, and sensitivity, this system lays the crucial technical groundwork for building practical POC diagnostic platforms [67] (Figure 5a). However, some microfluidic chips necessitate additional instrumentation, limiting their applicability for POCT use in settings with limited resources. Therefore, this highlights the ongoing demand for the creation of affordable and multi-channel detection-chip diagnostic solutions to address the high transmissibility of various EBOV subtypes.

Recent innovations by He and his team have led to the advancement of a CRISPR/Cas12a-based box system without the requirement for nucleic acid amplification. The system has achieved an LOD of 1 pM within 2 h [68] (Figure 5b). These advancements demonstrate the immense potential of combining CRISPR-Cas technology with microfluidic techniques, offering effective solutions for rapid and sensitive pathogen testing.

CRISPR technology has high sensitivity and specificity, but its application is still limited due to defects such as susceptibility to infection, high detection cost, complex sample handling, high miss rate, and so on. However, microfluidic technology, when combined with CRISPR, can complement and improve its detection capabilities, thus expanding its application in bioanalysis.

## 5. Smartphone Applications in Microfluidic Platforms

**Figure 6:**
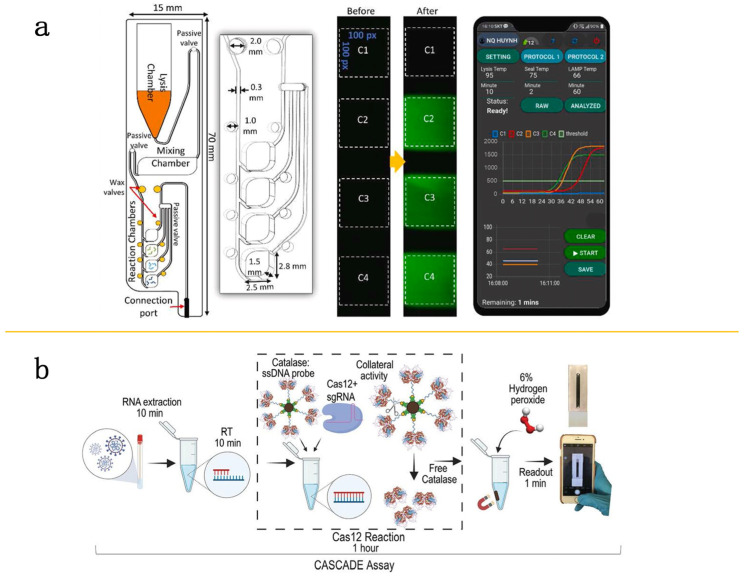
Microfluidic devices combined with smartphones. (**a**) Schematic representation of the smartphone-based microfluidic chip. The microfluidic chip features several components: a lysis chamber, a mixing chamber, reaction chambers, along with passive and wax valves (**left**). Chambers C2, C3, and C4 are pre-prepared with SARS-CoV-2 specific primers, whereas Chamber C1 serves as a negative control (**middle**). The real-time fluorescence signals and temperature profiles from each heater are transmitted to and displayed on a smartphone (**right**). Reproduced with permission [70]. Copyright 2021 Elsevier B.V. (**b**) Workflow of the cascade assay combined with smartphones. Viral RNA is isolated from swab samples and then reverse transcribed. It is incorporated into a Cas12 assay, which includes a catalase single-stranded DNA (CD) probe and oxygen. This setup produces a signal through the formation of oxygen bubbles, detectable in a microfluidic channel using a smartphone app. Reproduced with permission [71]. Copyright 2021 Wiley-VCH GmbH.

In recent years, researchers have increasingly turned to integrating microfluidic chip systems with smartphones to enable automated image acquisition without the need for traditional optical detectors. For example, Chen and colleagues designed a novel pipette tip featuring an elliptical profile in conjunction with a microfluidic device indicating a significant aspect ratio. This design can rapidly produce thousands of monodispersed droplets in less than 5 min for digital PCR analysis of the coronavirus. After PCR thermal cycling, the droplets are transferred to the center of a covered frame seal on a glass slide. Since the area of the entire monolayer suspension is about 1 cm^2^, it can be presented in the field of view of the smartphone. Subsequently, the results of PCR were recorded by placing a glass slide with droplets on the transilluminator and using the camera of the smartphone. Utilizing smartphone imaging technology, the system can detect positive droplets with an LOD of 3.8 copies/20 μL [69]. Similarly, Nguyen and their research group have introduced a compact microfluidic system that can execute multiple experimental tasks on a miniaturized chip. The outcomes of this device are processed through a microprocessor and shown via smartphone, which exhibits RT-LAMP results for clinical samples of SARS-CoV-2 with a detection limit of 20 copies/mL, demonstrating high sensitivity and accuracy [70] (Figure 6a). Yin and colleagues have successfully combined RPA with LAMP techniques on a microfluidic chip by 3D printing, utilizing smartphones to report and track test results. This integrated approach facilitates real-time data sharing and management [44]. Silva’s group has introduced a novel and amplification-free detection system that is smartphone-compatible and uses CRISPR-Cas-dependent enzymes for identification, capturing bubble signals produced by horseradish peroxidase within the microfluidic channels through smartphone imaging, achieving high-precision detection [71] (Figure 6b).

Similarly, Zhu and others created a rapid system for the extraction and detection of Zika, merging a microfluidic chip with chitosan-coated silica capillaries for enhanced smartphone-based detection. This microfluidic device can detect RNA at extremely low concentrations of 50 TU/ mL [72]. These studies show that combining microfluidic technology with smartphones not only enhances the convenience and accessibility of test methods but also significantly improves the sensitivity and accuracy of the analysis, providing effective solutions for rapid POC testing.

These results demonstrate that smartphone-based microfluidic systems can provide rapid and accurate detection without the need for complicated and expensive traditional analytical equipment or professional operation requirements. In addition, with their portability and universality, smartphone-based microfluidic systems can be integrated with other modules, which provides the possibility of expanding additional functions. However, compared to traditional testing equipment, smartphone-based systems lack sensitivity and accuracy. Additionally, the complexity of some integrated chips makes them difficult to popularize. At present, smartphone-based microfluidic systems are still in the proof-of-concept stage, making commercialization and technology transfer difficult.

## 6. AI-Assisted Microfluidic Technologies

In recent years, artificial intelligence and machine learning technologies have been widely applied in the field of testing. Similarly, these technologies have not only enhanced the accuracy and stability of the microfluidic detection system but also promoted the automation and integration of microfluidic devices. Sun and others created an innovative method that combines deep learning with microfluidic paper-based analytical devices (μPADs). This approach involves processing real-time PCR data through three deep neural network models: simple recurrent neural networks (RNNs), long short-term memory (LSTM) networks, and gated recurrent units (GRUs). Notably, the GRU layers enable precise prediction of PCR curve end-point values by the 20th cycle, achieving a mean absolute percentage error (MAPE) of 2.1% [73]. Wang et al. developed Fractal LAMP, an automated technique for detecting amplified DNA in droplets as small as subnanoliters, following the LAMP process without using labels. They utilized a computer vision algorithm that accurately identifies DNA amplification in these droplets by recognizing the fractal structures formed by LAMP byproducts and visible through brightfield microscopy. This method could reduce the costs associated with nucleic acid assays and sustain high quantitative precision across a concentration range spanning three orders of magnitude [74]. Solmaz and colleagues designed a smartphone app that uses machine learning classifiers to measure peroxide levels on colorimetric test strips. Images of these strips were captured using five different Android smartphones under seven varied lighting conditions. These images were then used to train binary classifiers (Least-Squares SVM) and multi-class classifiers (Random Forest). The results showed that the app could successfully identify changes in color on peroxide strips with an accuracy exceeding 90% [75]. Guo and colleagues introduced a smartphone-enabled platform for nucleic acid testing that enables multiplex DNA diagnosis of malaria. This platform incorporates deep learning algorithms into a mobile app, which autonomously classifies images from paper-based microfluidic diagnostic tests and offers localized decision support [76].

Artificial intelligence has given greater application potential to microfluidic technology, making it expected to become a powerful detection platform in the field of biomedical analysis. However, the application of artificial intelligence to microfluidic detection systems requires a large amount of training data to optimize the accuracy and stability of the system. The realization of cross-platform data cooperation is a major challenge for future development.

## 7. Paper-Based POC Testing Applications in Microfluidic Platforms

**Figure 7:**
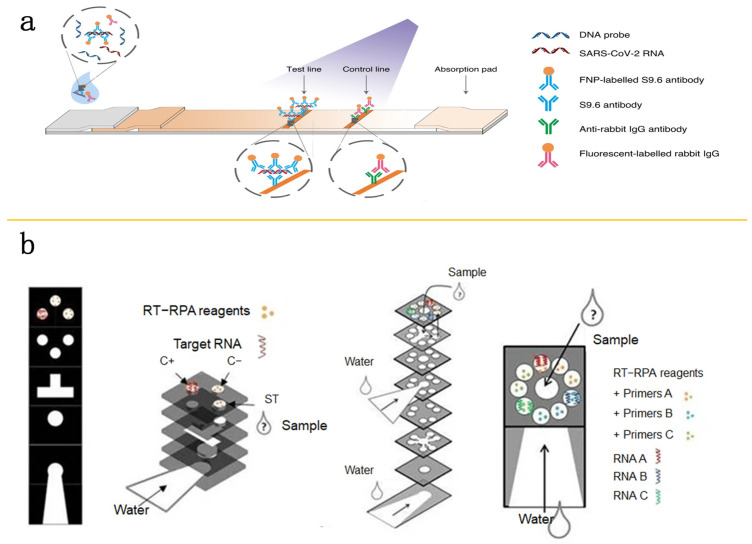
Paper-based POC microfluidic detection. (**a**) Schematic of HC-FIA assay. Reproduced with permission [77]. Copyright 2020 Springer Nature. (**b**) Multiplexed paper devices. Exploded diagram of the compacted device (**left**) and blueprints of the multi-analyte paper device (**right**). Reproduced with permission [79]. Copyright 2017 Springer Nature.

During the COVID-19 crisis, paper-based diagnostics have become increasingly significant. Owing to the suitability and low cost of paper materials, multiple paper-based SARS-CoV-2 detection tools have been fabricated, with the adoption of lateral flow assay (LFA) technology being especially prevalent. LFA is an established paper-based diagnostic approach that detects specific molecules on an absorbent membrane, resulting in the formation of test lines and control lines for the target molecules’ identification. This method offers rapid, simple, and portable visual or semi-quantitative analysis. In the development of COVID-19 diagnostic kits, researchers and manufacturers have devoted significant efforts and resources to LFA. For instance, Wang’s group has designed an amplification-free nucleic acid immunoassay capable of detecting the E and N genes of SARS-CoV-2 through fluorescence markers, achieving 100% sensitivity and 99% specificity [77] (Figure 7a).

EBOV has been categorized into five distinct types [78], with four (EBOV, SUDV, BDBV, TAFV) posing a serious hazard to human health, demanding the necessity for microfluidic technologies that can identify different EBOV strains. Magro’s team has fabricated a multiplex paper chip using RPA, which is capable of detecting multiple strains of EBOV. Their RPA-based paper microfluidic device underwent testing with patient samples from Guinea, demonstrating a 90% sensitivity when compared to RT-PCR results [79] (Figure 7b). Sanchita and others developed a multiplex paper-based PCR detection system that can identify four EBOV types [80]. These advancements suggest that paper-based POC testing technologies hold significant value in enhancing the accuracy, speed, and convenience of COVID-19 detection.

To further advance microfluidic diagnostics, additional technologies, such as optical waveguides, have been integrated to enable rapid and efficient detection. For instance, Du and colleagues have created a Sample Preparation Multiplexer (SPM) for Ebola virus detection, integrating an anti-resonant reflecting optical waveguide (ARROW) biosensor chip that achieves single-molecule detection with an LOD of 0.021 PFU/mL, all without the need for target amplification. This represents a two-order-of-magnitude improvement in RNA target capture efficiency and a tenfold increase in LOD compared to previous methods using streptavidin-coated beads [81]. Additionally, Cai’s research has demonstrated single-nucleic-acid fluorescence detection using a PDMS microfluidic device placed on silicon, completing tests with a detection limit of 0.2 PFU/mL and a dynamic range that spans 13 orders of magnitude in just 10 min; this method is significantly surpassing other non-amplification methods [82].

Paper-based diagnostic tools offer an accessible, low-cost solution for preliminary healthcare screenings at POC. However, this detection method also has some shortcomings. Firstly, visual interpretation of results, particularly in colorimetric lateral flow assays, can vary significantly. Secondly, the performance of these tests heavily relies on the durability and consistency of the equipment. Thirdly, variability across production batches poses a notable challenge in maintaining the tests’ repeatability. Consequently, enhancing the sensitivity and specificity of these paper-based diagnostics is crucial for their broader application.

## 8. Outlook

In essence, the primary aim of microfluidic technology is to enhance the performance of final products. However, current microfluidic-based systems cannot replace traditional methods. Although there has been notable progress in developing individual microfluidic components for disease detection devices, few technologies have transitioned into fully integrated devices offering clinical practical value. This is largely due to the absence of a clear roadmap for integration to build a coherent, fully functional integrated device. Consequently, it is challenging to improve the design decisions of individual components to promote the commercial application of microfluidic systems. Nevertheless, microfluidic systems hold promise as regular benchtop tools to aid clinicians and provide a broader range of reference information for disease monitoring. Here, we outline some key challenges towards achieving this practical objective:

(1) Developing modular microfluidic system components and procedures is essential for creating clinically meaningful integrated products.
(2) Formulating unified market supervision standards is necessary. Varying regulatory requirements across regions not only limit investor incentives but may also prevent technology from passing regulations and evolving into a clinical diagnostic system.
(3) Ensuring data connection to electronic medical record systems is crucial. With the increasing use of digital devices such as mobile phones, establishing remote data sharing platforms is very important.

Moving forward, attention should be given to the relationship between technology innovation and the commercialization process. As technology matures and new concepts emerge, microfluidics-based detection systems have great potential to be translated into clinically practical products.

## 9. Conclusions

Microfluidic technologies, combined with RT-PCR, LAMP, CRISPR, and mobile phone- and paper-based detection systems, have made significant processes in the rapid detection of infectious microbial pathogens. These advanced microfluidic platforms, which integrate molecular biology techniques, can efficiently and accurately detect a variety of pathogens, including SARS-CoV-2, Ebola virus, Zika virus, and dengue virus in a short amount of time. For instance, microfluidic RT-PCR devices can rapidly provide quantitative data on-site; the LAMP method is particularly well-suited for low-resource settings due to its simplicity and low equipment requirements; CRISPR technology offers high specificity in gene editing for pathogen identification; mobile-integrated detection systems enable instantaneous data sharing and analysis; and paper-based detection offers a cost-effective and scalable deployment option. Future development may focus on further improving detection sensitivity, reducing costs, streamlining operational procedures, and enhancing data processing and remote sharing capabilities, aiming to achieve broader geographic coverage and quicker response times. In summary, these advancements have not only optimized the process of pathogen detection but have also fortified a robust line of defense for global public health security.
